# Lost in translation: are doctors really answering patients’ questions about in vitro fertilization?

**DOI:** 10.1007/s10815-025-03538-x

**Published:** 2025-06-13

**Authors:** Naveena R. Daram, Malika L. Day, Molly J. Herr, Rose A. Maxwell, Meghan C. Ozcan

**Affiliations:** 1 Department of Obstetrics and Gynecology, Wright State University, 1 Wyoming St, Dayton, OH 45409 USA; 2 Boonshoft School of Medicine, Wright State University, Dayton, OH USA

**Keywords:** Social media, IVF, TikTok, Instagram, Reddit, Facebook

## Abstract

**Purpose:**

As social media grows as a health resource, IVF patients turn to Facebook and Reddit for guidance, while providers share educational content on TikTok and Instagram. This study examines the alignment between patient inquiries and provider content to identify gaps in digital fertility education.

**Methods:**

A cross-sectional qualitative analysis collected 50 top patient posts from five largest IVF Facebook and Reddit groups and 50 provider videos from Instagram and TikTok using popular IVF hashtags. Posts and videos were categorized into ten themes, engagement metrics analyzed, and provider content assessed for readability. Statistical analyses included chi-square tests for content distribution and independent *t*-tests and one-way ANOVA for readability differences (*p* < 0.05 considered significant).

**Results:**

Significant discrepancies were observed between patient inquiries and provider content. Patients most frequently inquired about medications, diagnostic testing, side effects, and emotional support—topics underrepresented in provider content. Providers disproportionately focused on procedural details (34.09% vs. 6.17%, *p* < 0.001). Readability analysis showed provider content exceeded the AMA-recommended 6th-grade reading level (range: 7.93–15.29). Despite misalignment, engagement with IVF-related content was high, with millions of views, likes, and comments across platforms.

**Conclusions:**

There is misalignment between what patients ask and what providers discuss on social media. Providers aiming content at patients should address patient priorities both online and in clinical practice while improving readability to enhance digital health literacy. Given the vast reach of social media, fertility specialists should consider establishing a presence to educate and engage with patients effectively.

**Supplementary Information:**

The online version contains supplementary material available at 10.1007/s10815-025-03538-x.

## Introduction

Social media has transformed how individuals seek and share health information, with an estimated 60–75% of adults turning to these platforms for medical advice and support [1, 2]. Patients often turn to platforms like Reddit and Facebook which serve as interactive spaces where patients pose questions, exchange personal experiences, and seek guidance from peers. Among reproductive health topics, in vitro fertilization (IVF) is a frequent subject of online discussion, with over 80% of infertility patients participating in social media forums and support groups [3, 4]. This growing role of social media as a supplementary source of medical information for patients reflects a shift in how individuals engage with their health and wellness.

Healthcare providers, recognizing the reach and influence of these platforms, have started to utilize social media to share educational content and address common health concerns. In one survey, 85% of healthcare providers agreed that social media can be an effective tool for education purposes [5]. Providers have increasingly adopted short-form video platforms like TikTok and Instagram to educate the public on medical topics, recognizing that their reach is often in the thousands to millions [6, 7]. However, despite the growing presence of medical professionals online, it remains unclear whether the educational content they provide aligns with the actual concerns and inquiries patients express on social media.

This study investigated the alignment between patient inquiries on social media forums and provider-generated educational content on short-form video platforms. By systematically comparing the themes of patient questions on Reddit and Facebook with healthcare provider content on TikTok and Instagram, this study aimed to identify gaps, assess engagement trends, and explore areas where provider content may better address patient needs. Understanding these discrepancies can provide insight into how healthcare professionals should refine their social media strategies to improve patient education and support in reproductive medicine.

## Materials and methods

### Study design

This study employed a cross-sectional analysis to compare patient queries and healthcare provider educational content on social media platforms. The goal was to assess how well provider content aligns with the questions and concerns expressed by patients seeking IVF.

### Time frame

No time frame restrictions were applied; instead, all publicly visible posts were included. Upon review, the posts spanned from 2017 to 2025. Data collection occurred between September 2024 and January 2025.

## Data sources

### Patient queries

Patient queries were collected from two major social media platforms—Reddit and Facebook. These platforms were selected for their distinct formats and extensive user bases. Both platforms have a group-based format, providing a space for community engagement and support within various health-related groups. They collectively offer a broad and active environment for patient discussions and inquiries.

- Reddit: Relevant posts were collected from IVF-related subreddits. To ensure analysis of active communities with sufficient user engagement, subreddits with fewer than 1000 members were excluded. The following subreddits met the inclusion criteria and were analyzed: r/IVF, r/Infertility_IVF, r/IVFAfterSuccess, r/IVFbabies, and r/IVFinfertility.
- Facebook: There were considerably more Facebook groups related to IVF compared to subreddits. To ensure a balanced comparison, the top five Facebook groups were selected based on membership size, aligning with the number of subreddits included in the study. Groups with < 1000 members were excluded.

### Provider educational content

Healthcare provider content was collected from short-form videos on TikTok and Instagram. These platforms were chosen due to their emphasis on concise, engaging content, which is particularly effective in capturing patients’ attention in today’s digital landscape. This format is ideal for representing how patients obtain educational material on social media. Only content created by verified healthcare professionals—such as physicians, nurse practitioners, and sonographers—was included. Whenever possible, the credentials of these professionals were verified by the reviewers to ensure the credibility of the information.

- TikTok and Instagram: After review of several IVF-related videos, the most frequently used hashtags were noted. Only hashtags explicitly containing “IVF” or “in vitro fertilization” were considered to ensure relevance to the study. The top five hashtags in the cohort were identified based on their popularity, as measured by Brand24, an AI-based hashtag tracking tool [8]. Popularity was determined by the volume of mentions associated with each hashtag. The top 5 hashtags that were identified and included in this study were #IVF, #invitrofertilization, #ivfjourney, #ivfprocess, #ivfsuccess. This method of using hashtags to search and analyze was based on and modified from prior studies [9–11].

Facebook and Reddit were chosen as the primary sources of patient-generated inquiries, as these platforms facilitate open-ended discussions where users explicitly ask questions and seek peer support. Conversely, Instagram and TikTok were selected to represent provider-generated content, as these platforms emphasize short-form video education, which healthcare professionals frequently use to communicate medical information. This study does not account for potential cross-platform interactions, such as patient questions in TikTok/Instagram comments or provider engagement in Facebook/Reddit forums.

## Data collection

### Extraction of patient queries

**Fig. 1:**
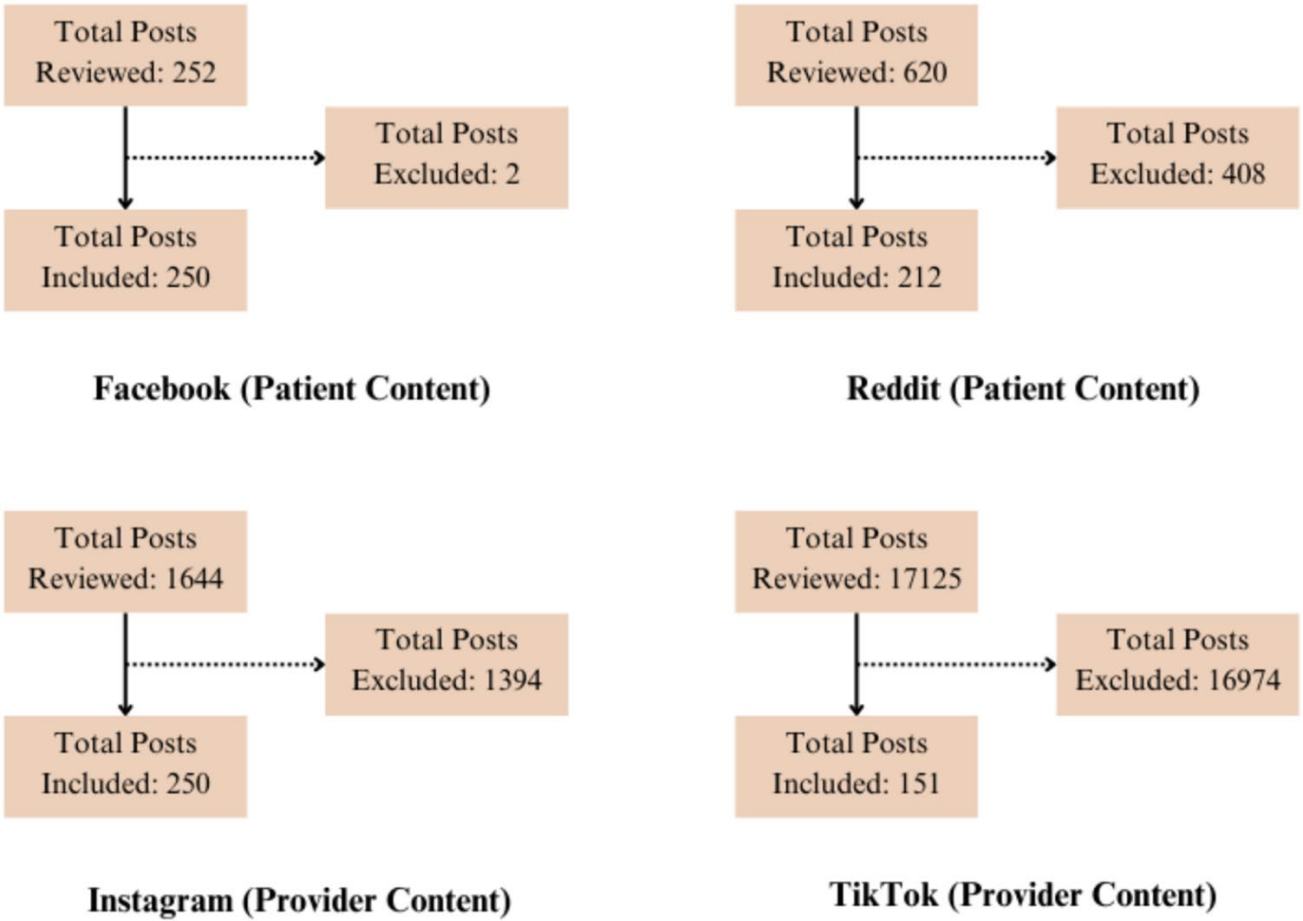
Flow diagram of posts and videos reviewed to reach sample

- Inclusion and exclusion criteria

- Inclusion criteria: Posts that specifically mentioned IVF-related concerns or questions were included. Only posts from adult users (age ≥ 18, assumed unless specified otherwise) were considered.
- Exclusion criteria: Posts not directly relevant to IVF were excluded.

- Sampling strategy

- Reddit: The posts were sorted by “Hot” which indicated the most popular postings sorted in chronological order. The top 50 posts were selected after confirming the inclusion and exclusion criteria, and this number was chosen based on prior studies conducted in other medical fields [10, 12]. In cases where 50 posts were not available for review in a group after meeting the inclusion and exclusion criteria, as many posts as possible were included in the analysis.
- Facebook: On Facebook, posts could be sorted by “Most Relevant,” “Newest Activity,” or “New Posts.” To capture posts with the highest engagement, we sorted by “Most Relevant.” A new Facebook profile was created to eliminate potential biases in the sorting algorithm, ensuring that the posts collected were genuinely those with the most user engagement. The top 50 posts were selected after confirming the inclusion and exclusion criteria.
- Figure 1 illustrates the number of posts reviewed before reaching the target sample size.

- Method of extraction: Patient queries were extracted using manual review by two reviewers. Once collected, both reviewers examined all posts to categorize them into 10 possible categories specified in Sect. 3.3. Posts were collected between September 2024 to January 2025.

### Extraction of provider educational content

- Inclusion and exclusion criteria

- Inclusion: Videos produced by verified healthcare providers that directly addressed IVF-related topics were included. Only videos with clear educational content relevant to IVF were selected.
- Exclusion: Videos lacking educational value or not related to IVF were excluded. Videos for which credentialing could not be verified were excluded.

- Sampling strategy

- The first 50 videos under each of the five most popular IVF-related hashtags (#IVF, #invitrofertilization, #ivfjourney, #ivfprocess, and #ivfsuccess) were selected for analysis on both Instagram and TikTok, following application of the inclusion and exclusion criteria. In cases where TikTok did not yield 50 eligible videos for a given hashtag, all available videos were included. The number of qualifying TikTok posts per hashtag ranged from 13 to 50.
- Figure 1 illustrates the number of posts reviewed before reaching the target sample size.

- Method of extraction

- Provider content was gathered through manual searches of the above hashtags. Two reviewers screened and selected videos from TikTok and Instagram, respectively. Once collected, both reviewers examined all videos to categorize them into 10 possible categories specified in Section 3.3. Posts were collected between September 2024 to January 2025.

### Categorization of queries and educational content

The following 10 categories were selected for patient queries and provider educational content based on the content and themes of the posts:

- Diagnostic testing
- Treatments
- Medications
- Procedural details
- Success rates
- Side effects and risks
- Costs and finances
- Ethical and legal issues
- Emotional support
- Other

Some patient queries and provider videos addressed multiple thematic categories. In such cases, posts and videos were assigned to all relevant categories to accurately capture the full scope of discussion. For example, a post asking about “IVF medication side effects and their impact on success rates” was categorized under both “Medications” and “Success Rates.” This approach was necessary because IVF-related concerns are multifaceted, and assigning a post to a single category would have resulted in loss of important contextual information. Each post was independently reviewed and categorized by two researchers, who reached a consensus on the final categorization.

The “Other” category encompassed topics that did not clearly align with the nine predefined categories. Patient-generated content in this category included discussions such as specific IVF clinic recommendations, inquiries about providers, and comparisons of IVF regulations between the United States and other countries. Provider-generated content categorized as “Other” included promotions of supplements or alternative therapies such as acupuncture, explanations of embryo development in the laboratory, and discussions on specific genetic testing. Due to the limited frequency and diverse nature of these topics, they could not be grouped into a separate, distinct category and were therefore classified under “Other.”

## Data analysis

### Thematic analysis

- Both patient queries and provider educational content across the four social media platforms were divided into the 10 categories previously mentioned.
- The number of posts and videos under each category, separated by provider content and patient query, were recorded to include total count.
- Table 1 presents the percentage distribution of patient-generated and provider-generated content across the 10 thematic categories, stratified by social media platforms. Percentages were calculated using the formula: percentage = (posts in category for a platform ÷ total posts in that platform) × 100. The total across all categories sums to 100% per platform, allowing for a standardized comparison of content distribution. Using raw post counts alone would not allow for direct comparisons, as the total number of posts differed across platforms. By converting to percentages, we were able to compare how frequently different topics are discussed within each platform while accounting for differences in sample sizes.
- Chi square test was used to compare the distribution of themes between patient and provider content. *p* < 0.05 was considered to be statistically significant.

**Table 1:** Distribution of patient and provider content by category across social media platforms

Category	Patient			Provider			*p*^a^	Over or under representation?
	Facebook (*n* = 250)	Reddit (*n* = 212)	Combined (*n* = 462)	Instagram (*n* = 250)	Tiktok (*n* = 151)	Combined (*n* = 401)		
Diagnostic testing	7.20% (*n* = 18)	14.70% (*n* = 30)	10.57% (*n* = 48)	7.20% (*n* = 18)	2.74% (*n* = 4)	5.56% (*n* = 22)	0.009	Under
Treatment options	10.40% (*n* = 26)	8.33% (*n* = 17)	9.47% (*n* = 43)	21.20% (*n* = 53)	7.53% (*n* = 11)	16.16% (*n* = 64)	0.003	Over
Medication inquiries	10.00% (*n* = 25)	15.69% (*n* = 32)	12.55% (*n* = 57)	3.20% (*n* = 8)	2.74% (*n* = 4)	3.03% (*n* = 12)	< 0.001	Under
Procedural details	4.80% (*n* = 12)	7.84% (*n* = 16)	6.17% (*n* = 28)	17.60% (*n* = 44)	62.33% (*n* = 91)	34.09% (*n* = 135)	< 0.001	Over
Success rates	28.80% (*n* = 72)	11.76% (*n* = 24)	21.15% (*n* = 96)	22.80% (*n* = 57)	6.85% (*n* = 10)	16.92% (*n* = 67)	0.135	No difference
Side effects and risks	10.80% (*n* = 27)	5.88% (*n* = 12)	8.59% (*n* = 39)	4.00% (*n* = 10)	1.37% (*n* = 2)	3.03% (*n* = 12)	< 0.001	Under
Costs and financial concerns	3.60% (*n* = 9)	2.94% (*n* = 6)	3.30% (*n* = 15)	2.00% (*n* = 5)	1.37% (*n* = 2)	1.77% (*n* = 7)	0.166	No difference
Ethical and legal issues	0% (*n* = 0)	0% (*n* = 0)	0% (*n* = 0)	5.60% (*n* = 14)	3.42% (*n* = 5)	4.80% (*n* = 19)	< 0.001	Over
Emotional support	12.80% (*n* = 32)	20.59% (*n* = 42)	16.30% (*n* = 74)	4.40% (*n* = 11)	0.68% (*n* = 1)	3.03% (*n* = 12)	< 0.001	Under
Other	11.60% (*n* = 29)	12.25% (*n* = 25)	11.89% (*n* = 54)	12.20% (*n* = 30)	10.96% (*n* = 16)	11.62% (*n* = 46)	0.939	No difference

Data is displayed as percentage distribution (%), with n representing the number of posts or videos within each respective category. Percent distribution was calculated as: percentage = (total posts in that platform ÷ posts in category for a platform) × 100

^a^: p represents the statistical comparison between patient-generated content (Facebook + Reddit) and provider-generated content (Instagram + TikTok) for each category

Overrepresentation indicates that providers disproportionately covered a category compared to patient queries; underrepresentation suggests a relative lack of provider content on that topic; no difference indicates alignment between patient and provider content

### Engagement analysis

- The number of members of each subreddit group and Facebook group were recorded.
- The number of likes/upvotes and comments for each video and post were collected to analyze engagement for both patient queries and provider content. For videos, the number of views were also recorded.
- This study could not directly compare engagement across categories because each social media platform uses different algorithms to determine content visibility and interaction, which can bias engagement metrics. Differences in user preferences and platform popularity may also further skew engagement statistics. Additionally, posts on Facebook and Reddit do not display view counts, while videos on TikTok and Instagram do, making direct comparisons of engagement not accurate. As a result, engagement data was reported only descriptively in Tables 2 and 3.

**Table 2:** Engagement by platform

Social media platform	Total posts/videos	Total likes/upvotes	Total comments	Total views
Facebook	250	3511	3808	N/A
Reddit	212	1344	2100	N/A
Instagram	250	2205965	28169	132158275
TikTok	151	5051336	71428	96272047

**Table 3:** Engagement by content category

Category	Total posts/videos	Total likes/upvotes	Total comments	Total views
Diagnostic testing	70	687,504	11,840	17,534,152
Patient inquiries	48	141	293	N/A
Provider content	22	687,363	11,547	17,534,152
Treatment options	107	568,143	4,295	28,868,990
Patient inquiries	43	125	343	N/A
Provider content	64	568,018	3,952	28,868,990
Medication inquiries	69	36,270	930	3,500,575
Patient inquiries	57	76	381	N/A
Provider content	12	36,194	549	3,500,575
Procedural details	163	4,292,798	56,288	100,465,403
Patient inquiries	28	115	444	N/A
Provider content	135	4,292,683	55,844	100,465,403
Success rates	163	925,972	11,830	40,728,582
Patient inquiries	96	2,713	1913	N/A
Provider content	67	923,259	9,917	40,728,582
Side effects and risks	51	133,977	2,243	4,122,000
Patient inquiries	39	108	526	N/A
Provider content	12	133,869	1717	4,122,000
Cost and financial concerns	22	82,381	2204	3,452,465
Patient inquiries	15	88	392	N/A
Provider content	7	82,293	1812	3,452,465
Ethical and legal issues	19	121,469	8052	5,101,674
Patient inquiries	0	0	0	N/A
Provider content	19	121,469	8052	5,101,674
Emotional support	86	27,386	1130	1,034,673
Patient inquiries	74	1299	864	N/A
Provider content	12	26,087	266	1,034,673
Other	100	386,256	6,793	23,621,808
Patient inquiries	54	190	752	N/A
Provider content	46	386,066	6,041	23,621,808

Average views were recorded only where applicable. Not all of the content in these categories was through videos

### Reading level analysis

- Healthcare professionals’ videos from Instagram and TikTok were transcribed using the Voice Typing tool in Google Docs. The transcribed text was then analyzed with a readability calculator that integrates eight different readability formulas to determine an average reading level [13].
- Only provider-generated content was analyzed for readability, as the goal was to assess whether patients could understand the educational content provided by healthcare professionals. Evaluating the readability of patient-generated inquiries was deemed unnecessary, as these posts reflect personal experiences and questions rather than educational material.
- Table 3 presents the reading level of provider content across the 10 categories, separated by social media platform (Instagram vs. TikTok). Independent *t*-tests were conducted to determine whether significant differences exist in readability between platforms and one-way ANOVA across all content categories. *p* < 0.05 was considered to be statistically significant.

## Ethical considerations

This study was deemed exempt by our institution’s review board (IRB-2025–769) because only publicly available data was used and there was no direct interaction with subjects. Patient posts and provider videos were anonymized where possible to protect privacy.

## Results

### Thematic analysis

**Fig. 2:**
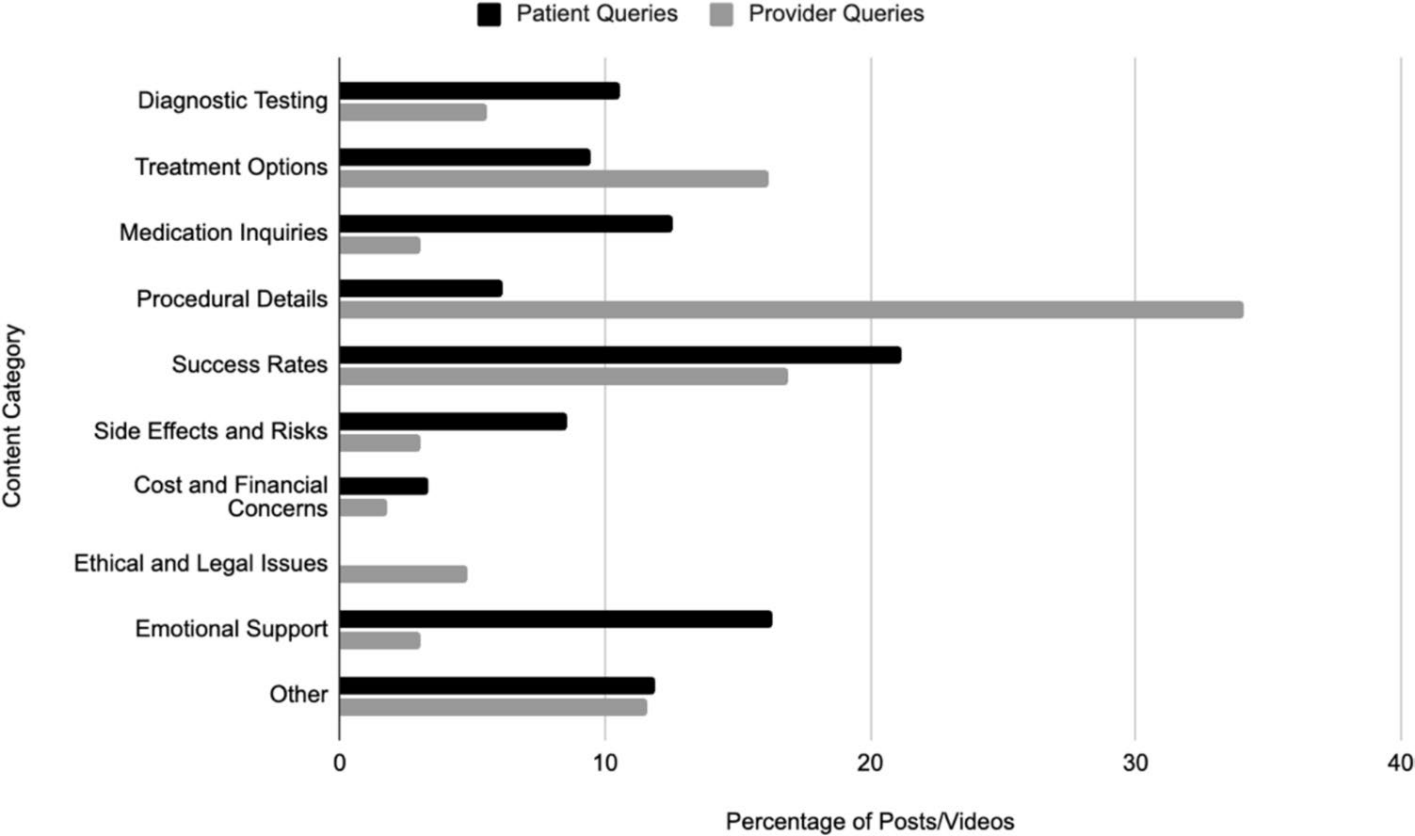
Visual comparison of patient queries vs. provider content across categories

The distribution of patient-generated and provider-generated content across thematic categories is presented in Table 1 and visualized in Fig. 2. Statistically significant differences were observed between the topics emphasized by patients and providers, with notable disparities in the representation of various IVF-related themes.

Provider content was significantly more focused on procedural details, with 34.09% of provider-generated posts discussing procedural aspects of IVF compared to 6.17% of patient queries (*p* < 0.001). Treatment options were also more frequently covered by providers than patients (16.16% vs. 9.47%, *p* = 0.003), as were ethical and legal issues, which appeared in 4.80% of provider content but were entirely absent from patient queries (*p* < 0.001).

Conversely, several categories were significantly more common in patient-generated content than in provider posts. Medication-related questions accounted for 12.55% of patient posts but only 3.03% of provider content (*p* < 0.001). Diagnostic testing was similarly discussed more frequently by patients (10.57%) than by providers (5.56%) (*p* = 0.009). Emotional support was another underrepresented category in provider content, with only 3.03% of provider videos addressing this topic compared to 16.30% of patient queries (*p* < 0.001).

Other categories showed no statistically significant difference in representation between patient and provider content. Discussions about success rates were similar between the two groups (*p* = 0.135), as were concerns regarding costs and financial issues (*p* = 0.166). The “Other” category also showed no significant difference in distribution (*p* = 0.939).

## Engagement statistics

We analyzed five Facebook groups with a total of 213,900 members and five Reddit groups with 55,769 members, resulting in a combined engagement of 269,669 individuals from whom patient inquiries were sampled from. On TikTok, the five selected hashtags yielded 939,421 videos, while the five Instagram hashtags generated 1644 videos, totaling 941,065 IVF-related videos. Table 2 presents the total number of likes, upvotes, comments, and views by platform. Across all platforms, the total engagement derived from 863 posts and videos was: 7,262,156 likes/upvotes, 105,505 comments, and 228,430,322 video views.

Table 3 presents engagement metrics for patient-generated and provider-generated content across content categories. Within patient-generated inquiries, the emotional support category generated the largest number of posts (74), which received 1299 likes and 864 comments, whereas the ethical and legal issues category had no patient-generated posts. Within provider-generated content, the procedural details category had the highest number of posts (135), receiving 4,292,683 likes, 55,844 comments, and 100,465,403 views. Costs and financial concerns category had the lowest number of posts (8), receiving 82,293 likes, 1812 comments, and 3,452,465 views.

## Reading level analysis

The average reading level of provider-generated content on Instagram and TikTok across categories is presented in Table 4. Among the category comparisons between platforms, only success rates showed a significant difference in reading level (*p* = 0.04), indicating a measurable discrepancy in provider content complexity across the two social media platforms. Procedural details and emotional support approached significance (*p* = 0.07), suggesting potential differences that did not reach statistical significance. When comparing reading levels across all content categories, no significant difference was found (*p* = 0.105), indicating that readability was generally consistent across topics.

**Table 4:** Reading level of provider content on social media

Category	Reading level			*p*	
	Instagram	TikTok	Combined	Instagram vs. TikTok	Across categories
Diagnostic testing	11.31 ± 8.04 (*n* = 18)	8.70 ± 2.74 (*n* = 4)	10.84 ± 7.38 (*n* = 22)	0.76	0.105
Treatment options	16.35 ± 34.70 (*n* = 53)	10.18 ± 2.79 (*n* = 11)	15.29 ± 31.63 (*n* = 64)	0.53	
Medication inquiries	8.51 ± 2.58 (*n* = 8)	10.28 ± 1.95 (*n* = 4)	9.10 ± 2.46 (*n* = 12)	0.23	
Procedural details	9.52 ± 2.45 (*n* = 44)	8.97 ± 2.59 (*n* = 91)	9.15 ± 2.55 (*n* = 135)	0.07	
Success rates	7.37 ± 2.45 (*n* = 57)	11.14 ± 2.33 (*n* = 10)	7.93 ± 2.77 (*n* = 67)	0.04	
Side effects and risks	12.20 ± 1.84 (*n* = 10)	11.23 ± 1.97 (*n* = 2)	12.04 ± 1.81 (*n* = 12)	0.67	
Costs and financial concerns	8.86 ± 3.59 (*n* = 5)	7.84 ± 0.73 (*n* = 2)	8.57 ± 2.99 (*n* = 7)	0.76	
Ethical and legal issues	11.36 ± 3.87 (*n* = 14)	10.72 ± 1.25 (*n* = 5)	11.19 ± 3.35 (*n* = 19)	0.42	
Emotional support	11.56 3.36 (*n* = 11)	4.93 ± 0 (*n* = 1)	11.01 ± 3.73 (*n* = 12)	0.07	
Other	7.31 ± 2.86 (*n* = 30)	10.58 ± 2.75 (*n* = 16)	8.45 ± 3.20 (*n* = 46)	0.09	

## Discussion

This study reveals a misalignment between the topics patients seek information about and the content providers create on social media. Patients frequently inquired about medications, diagnostic testing, emotional support, and side effects/risks, yet these topics were underrepresented in provider-generated content. Instead, providers overrepresented procedural details, suggesting that the current landscape of online fertility education does not fully reflect patient priorities. These findings align with prior research indicating that IVF patients use social media primarily for emotional support and experiential sharing [14]. The psychological burden of infertility is substantial, with research suggesting that infertile patients experience a major depression almost three times as much as fertile patients [15]. The underrepresentation of emotional support in provider content highlights a critical gap in addressing these psychosocial concerns. Interestingly, financial concerns, a well-documented barrier to IVF access, were evenly represented between patients and providers [16]. This suggests that cost-related discussions may already be occurring in clinical settings, reducing the need for further online inquiries.

The engagement surrounding IVF-related content on social media is substantial, accumulating millions of views far exceeding traditional academic publications, emphasizing the power of social media as a patient education tool. These findings align with prior research highlighting the increasing presence of healthcare providers on social media [17]. For fertility specialists engaging online, defining their intended audience is essential. If targeting patients, content should align with patient needs; if aimed at colleagues, educational strategies may differ. A limitation of this study is the inability to determine whether providers were creating content for patients or for peer education, which may partly explain the observed misalignment.

The readability analysis suggests another potential barrier to patient education, with provider-generated content displaying variability, ranging from 7.93 to 15.29. Despite this range, all transcribed videos exceeded the 6 th-grade reading level recommended by the American Medical Association for patient education materials, potentially limiting accessibility for some patients [18, 19]. Notably, a significant readability difference was observed between TikTok and Instagram in the success rates category, suggesting possible platform-based variations in content complexity. However, readability remained largely consistent across other categories. Given the growing reliance on social media for health information, ensuring that content is both comprehensible and accessible is critical to minimizing digital health disparities.

This study has several strengths. It is the first to systematically compare patient-generated inquiries and provider-generated content across multiple social media platforms regarding infertility content. It involves a comprehensive strategy that evaluates how fertility information is disseminated online, integrating thematic analysis, engagement metrics, and readability assessments. The inclusion of multiple platforms (Facebook, Reddit, TikTok, and Instagram) allows for a broad assessment of how discussions unfold in various digital spaces. However, limitations include the exclusion of provider engagement in comment sections, potential content visibility biases due to social media algorithms, and the restriction to English-language content, limiting generalizability to non-English-speaking populations. Additionally, as a cross-sectional analysis, this study captures only a single time window rather than evolving trends in patient-provider interactions. Future research should focus on the impact of targeted interventions to improve alignment between patient inquiries and provider education and explore the role of digital health literacy in accessing and understanding IVF-related content.

From a clinical perspective, these findings highlight the need for fertility specialists to incorporate more patient-centered education into their communication strategies. Incorporating the topics patients frequently inquire about on social media into initial fertility consultations can strengthen patient-provider communication and better support individuals navigating IVF. Research shows that patients who feel informed and supported are more likely to adhere to treatment recommendations and report higher satisfaction with care [20, 21]. Ensuring that provider content directly addresses common patient concerns—particularly medications, side effects, and emotional support—could help reduce patient anxiety and improve IVF experiences. Additionally, simplifying content for improved comprehension could enhance digital health literacy and ensure that critical information is accessible to all patients.

## Conclusion

This study reveals a significant misalignment between patient inquiries and provider-generated content on social media, with providers emphasizing procedural aspects of IVF while patients seek more information on medications, diagnostic testing, and emotional support. The findings highlight the need for healthcare professionals to tailor their social media content to better address patient concerns and improve accessibility through clearer, more patient-centered communication. Future efforts should focus on optimizing digital health education strategies to enhance patient engagement and support informed decision-making in reproductive medicine.

## Supplementary Information

Below is the link to the electronic supplementary material.

TikTok Data (XLSX 155 kb)

Reddit Data (XLSX 120 kb)

Instagram Data (XLSX 44.6 kb)

Global Data Set (XLSX 102 kb)

Facebook Data (XLSX 23.0 kb)

## Data Availability

Our datasets are provided as excel files in supplementary material.
